# Effects of stress on sleep quality: multiple mediating effects of rumination and social anxiety

**DOI:** 10.1186/s41155-024-00294-2

**Published:** 2024-03-18

**Authors:** Jun Zhang, Xiaowen Li, Zhenxing Tang, Shungui Xiang, Yin Tang, Wenxin Hu, Chenchen Tan, Xin Wang

**Affiliations:** 1 Sehan University, College of Education, Jeollanam-Do, 1113 Green Road Samho Eup, Yeongam County, 650106 Republic of Korea; 2 Zhejiang Tourism College, Hangzhou, 310000 China; 3 School of Educational Sciences, Huangshan University, Huangshan, 245021 China

**Keywords:** Stress, Sleep quality, Social anxiety, Rumination

## Abstract

**Background:**

In contemporary society, with the accelerated pace of work and life, more and more people feel different degrees of stress. Long-term stress may not only lead to insomnia, but also to mental health problems (e.g., anxiety and depression), which has a significant impact on people's quality of life and mental health.

**Objective:**

This study primarily investigates the mechanism through which stress affects sleep quality among college students.

**Methods:**

We conducted research on 1653 Chinese college students using four scales with high reliability and validity: stress, the Pittsburgh Sleep Quality Index, social anxiety, and rumination.

**Results:**

The study found: (1) Stress can significantly and positively predict sleep quality and rumination; (2) Rumination can positively predict social anxiety; (3) Social anxiety can positively predict sleep quality; (4) Stress can affect sleep quality through social anxiety and rumination separately, and stress can also affect sleep quality through the chained mediation of rumination and social anxiety.

**Conclusion:**

This study reveals the relationship and mechanisms between stress and sleep quality. It not only deepens the research on the impact of stress on sleep quality but also provides theoretical support and new methods for mental health professionals to help clients improve their sleep quality. In practice, in addition to using some common psychological intervention methods to help individuals reduce stress, we should pay more attention to how to help clients reduce rumination and social anxiety, This is significant in improving the quality of an individual's sleep.

## Background

Stress refers to a series of physiological and behavioral changes that occur in an individual during the process of experiencing negative emotions. Individuals strive to adapt by manipulating the environment to alter the impact caused by the source of stress (Tao, 2006). Studies have shown that, with the rapid development of socio-economics and urbanization, the level of stress faced by Chinese people has generally increased. Particularly in work environments, family responsibilities, and social expectations, there has been a significant increase in stress experienced by Chinese individuals (Li & Wang, 2020).

Sleep quality typically refers to the depth and restorative nature of sleep, including the ease of falling asleep, the duration of sleep, the number of awakenings during the night, and the feeling upon waking in the morning (Liu et al., 2023). Poor sleep quality can lead to a range of health issues. For instance, long-term sleep disorders are associated with cardiovascular diseases (Doolin et al., 2018; Irwin, 2015), metabolic syndrome, digestive problems, decreased immune function, and cognitive impairments (Barger et al., 2006). An individual maintaining a lower quality of sleep over an extended period can further harm their mental health (Gardani et al., 2021).

Researchers conducted empirical analysis on sleep quality and stress among different populations aged 18–61 (including university students, employed individuals, unemployed individuals, and retirees). Through univariate analysis, results showed a significant negative correlation between stress and sleep quality. Sleep quality is at least partly related to pre-sleep stress, waking times from sleep, and subjective health (Bai et al., 2022; Kerstedt et al., 2012; Lund et al., 2010; Zhao et al., 2021). Many researchers have also confirmed that stress is a significant factor in reducing sleep quality (Li et al., 2019; Zhang et al., 2021), indicating that lower stress leads to better sleep quality (Grant & Benham, 2019; Kumari et al., 2020; Siddique et al., 2021).

Rumination is a process of continuously and repetitively thinking about one's own negative emotions, their causes, and consequences, and is often closely related to the individual's experienced stress level and social anxiety. For instance, when considering buying a house, intuitive thinking might lead you to focus more on the size of the house, its location, and price factors. However, when employing rumination in thinking about buying a house, you might consider whether the house meets our needs for the next ten years, whether the embryonic educational resources, medical conditions, and housing security policies of the house meet our expectations. We even need to consider what payment method to use and whether we have the sustained ability to repay. At this point, the factors we consider become endless, leading to hesitation and inner turmoil. Researchers have found that an individual's continuous rumination can intensify their negative emotional response to stress, thereby increasing the perception of psychological stress. This relationship is particularly evident in social scenarios because rumination often leads to negative interpretations of one's past social activities, thereby increasing the risk of social anxiety (Zawadzki, 2015). Furthermore, studies have found that there is a significant positive correlation between rumination and social anxiety. Rumination not only intensifies an individual's anxiety about social events but may also lead to avoidance behavior in future social interactions. This pattern of thinking can make individuals more sensitive and vulnerable in social situations, leading to increased levels of social anxiety (Kocovski & Rector, 2007). However, few have assessed the role of rumination and social anxiety in the relationship between stress and sleep quality.

Some studies suggest that it is necessary to assess the deeper mechanisms by which stress affects sleep quality (Kang et al., 2020). This can better help us to improve sleep and life quality effectively through interventions aimed at reducing stress (Akerstedt et al., 2012; McLean et al., 2021; Zhang et al., 2021). Based on this, this study proposes Hypothesis 1: University student stress can negatively predict sleep quality.

### The mediating role of social anxiety

Social anxiety refers to the anxiety symptoms that arise in individuals in social situations due to the fear of negative evaluation by others (Greenberg & Reyes, 2022). Individuals may worry that their behavior or performance will lead to negative evaluations, creating significant discomfort and anxiety. Symptoms of social anxiety include accelerated heartbeat, sweating, trembling, dry mouth, confused thinking, and avoidance of social activities. Long-term social anxiety not only affects an individual's social functioning and quality of life but can also lead to other mental health issues, such as depression and substance abuse (Stein & Stein, 2008).

The Stress Vulnerability Model suggests that an individual's susceptibility to stress may increase the risk of social anxiety, with a significant correlation between the two (Tillfors et al., 2013). Stress or major life changes can affect an individual’s cognitive level, making them more likely to have negative expectations and excessive focus on their performance in social situations (Kocovski et al., 2007; Rapee, 2001).

The cognitive model of stress posits that the continuous worry and overthinking of individuals with social anxiety may lead to difficulty falling asleep and sleep interruptions, thereby affecting sleep quality (Alfano et al., 2009; Friedman et al., 2005). This can exacerbate symptoms of depression, increase body fat (Haidar et al., 2018), lead to poor self-perception (Burke et al., 2017), and amplify their perception of environmental threats (Fadardi et al., 2023), thus hindering their overall development (Gong et al., 2023). Researchers have conducted surveys on the sleep quality of children and adolescents aged 6–18 in Beijing, China (Lima et al., 2020), and 238 stroke patients (Zhao et al., 2021), confirming that social anxiety is associated with sleep quality, more frequent nighttime awakenings, and longer sleep onset times (Buckner et al., 2008; Feng et al., 2019; Monti & Monti, 2000). Based on this, this study proposes Hypothesis 2: Social anxiety mediates the relationship between university student stress and sleep quality.

### The mediating role of rumination

The Response Style Theory posits that individuals typically adopt one of the following coping styles after being subjected to stress stimuli: rumination, problem-solving, and attention diversion. Rumination is a repetitive and ineffective thinking pattern that usually focuses on one's negative experiences and emotions, as well as the causes and consequences of these experiences (Moulds et al., 2020; Nolen-Hoeksema et al., 2008; Robinson & Alloy, 2003). This mode of thinking is often self-focused and can manifest as constant review, analysis, and reflection on past unpleasant events (Watkins, 2008). Rumination has a significant negative impact on an individual's mental and physical health and is considered an important maintenance factor for symptoms of depression and anxiety (Lyubomirsky et al., 2015). Prolonged rumination not only exacerbates and prolongs depressive moods but can also lead to impaired cognitive function, reduced social functioning, and decreased life satisfaction (Nolen-Hoeksema, 2000).

Studies have shown significant gender differences in rumination, with males tending to rely on distractions and other techniques to reduce negative emotions (Jose & Brown, 2008), whereas females exhibit more pronounced rumination than males, and their risk of depression is about twice that of males (Nolen-Hoeksema & Puryear-Keita, 2003).

Stress is a significant contributing factor to rumination. According to Cognitive Vulnerability Theory, when individuals face stress, their cognitive style tends to exhibit rumination, which makes them more prone to negative emotions and psychological health issues (Abela & Hankin, 2008). Research has found that many stressful events significantly increase the frequency and intensity of rumination (Michl et al., 2013). For example, stressors like work stress interpersonal relationship issues, and health problems have been found to be significantly associated with rumination (Zoccola et al., 2008). Rumination not only intensifies the negative impact of stress but can also lead to the development of depression, anxiety, and other mental health issues (Nolen-Hoeksema, 2000).

Rumination has a significant negative impact on sleep quality. According to the cognitive-behavioral model, rumination disrupts the normal sleep process by increasing cognitive arousal and emotional activation (Harvey, 2002). The persistent and repetitive nature of rumination makes it difficult for individuals to disengage from negative thinking, thereby increasing the difficulty in falling asleep and maintaining good sleep quality (Guastella & Moulds, 2007). Indeed, rumination has been associated with various sleep problems, including difficulty falling asleep, frequent awakenings during the night, early morning awakening, decreased sleep quality, and sleep disturbances (Pillai et al., 2014; Thomsen et al., 2003). Based on this, this study proposes Hypothesis 3: Rumination mediates the relationship between university student stress and sleep quality.

### The chain mediating role of rumination ang social anxiety

Rumination is considered a key maintenance factor for social anxiety, significantly impacting it (Kocovski et al., 2005). This thinking pattern leads individuals to excessively focus on their performance in social situations and potential negative evaluations, thereby increasing the degree of social anxiety (Morrison & Heimberg, 2013). Research has also found that rumination is positively correlated with the severity of social anxiety symptoms; that is, the more frequent the rumination, the more severe the symptoms of social anxiety (Brozovich & Heimberg, 2013). Additionally, rumination may affect the social skills and behaviors of individuals with social anxiety, leading to more social avoidance and social functioning impairments (Rood et al., 2010). Combining these research findings, this study proposes Hypothesis 4: Rumination and social anxiety act as a chained mediating factor between stress and sleep quality.

## Methods

### Participants

The participants for this study were recruited from undergraduates and graduates of Anhui Normal University, Qinghai Minzu University, Sichuan University of Light Chemical Engineering, and Hefei Normal University. In selecting human participants, we strictly adhered to the relevant clauses of the Declaration of Helsinki. A total of 1675 questionnaires were distributed, and 1653 valid questionnaires were retrieved, making the response rate 98.7%. Among the participants, there were 602 males (36.4%) and 1051 females (63.6%). 76.7% of the participants were from urban areas, while 23.3% were from rural areas. The average age of the participants was 19.41 ± 1.174 years, with the average age for males being 19.59 ± 1.187 years and for females being 19.31 ± 1.154 years. Before conducting the survey, we explained the purpose and method of our questionnaire to the recruited participants, ensuring each participant was fully informed. Informed consent forms were signed by both parties, and each participant received a financial compensation of 100 RMB. The ethics committees of Anhui Normal University, Qinghai Minzu University, Sichuan University of Light Chemical Engineering, and Hefei Normal University all approved the implementation of this project.

### Research tools

#### Stress scale for college students (SSCS)

We used the Stress Scale for College Students developed by Li and Mei (2002) to measure the stress level of college students. This scale consists of 30 items across three factors (academic disturbances, personal disturbances, and negative life events), scored on a Likert 4-point scale. The options for each item are: 0 for "no stress", 1 for "mild stress", 2 for "moderate stress", and 3 for "severe stress". Higher scores indicate greater stress experienced at the current stage. The scale has good reliability and validity, with a Cronbach's alpha coefficient of 0.91 and a test–retest reliability of 0.78 (Li & Mei, 2002).

#### Pittsburgh sleep quality index (PSQI)

We used the Pittsburgh Sleep Quality Index, developed by Buysse et al.to measure the sleep quality of participants over the past month. This scale consists of 24 items across seven factors (subjective sleep quality, sleep latency, sleep duration, habitual sleep efficiency, sleep disturbances, use of sleeping medication, and daytime dysfunction), with two items being non-scoring (Yan et al., 2010). It uses a Likert 4-point scoring system, with higher scores indicating poorer sleep quality. The scale has a Cronbach's alpha coefficient of 0.784 and a validity of 0.85 (Liu et al., 2023).

#### Social anxiety scale

We used the Social Anxiety Scale revised by Fenigstein et al. This scale comprises six items in a single factor, with each item offering four options: 0 for "not at all like me", 1 for "a little like me", 2 for "somewhat like me", and 3 for "very much like me". The item stating "I am at ease talking to strangers" is scored in reverse, while the rest are scored directly. Higher scores indicate higher levels of social anxiety. The overall Cronbach's alpha coefficient of the scale is 0.79, with a test-retest reliability of 0.73 (Lindwall & Magnus, 2004).

#### Ruminative responses scale (RRS)

The measurement of rumination was based on the Ruminative Responses Scale, revised by Han and Yang (2009) from the scale developed by Nolen-Hoeksema. The revised scale consists of 22 items across three factors (symptom-focused rumination, reflective pondering, and compulsive meditation). It uses a Likert 4-point scoring system, with options ranging from 1 for "never think about depressing things" to 4 for "always think about depressing things". The overall Cronbach's alpha coefficient of the scale is 0.90, with a test–retest reliability of 0.82 (Han & Yang, 2009).

## Results

### Data analysis

We utilized SPSS 25.0 software to analyze the mean, standard deviation, and Pearson correlation coefficients of stress, rumination, social anxiety, and sleep quality. For confirmatory factor analysis, we used Mplus 7.0 software to test the internal structure validity of the questionnaire and employed the maximum likelihood estimation method to handle missing questionnaire data. The research was carried out in two steps: First, to verify whether the independent mediating roles of rumination and social anxiety in the relationship between college students' stress and sleep quality were valid. Second, to confirm the chained mediating effect of rumination and social anxiety between college students' stress and sleep quality. Initially, we built a direct effect model to assess the direct effect of stress on sleep quality. Subsequently, we included rumination and social anxiety as mediating variables in the model of stress and sleep quality to construct a chained mediating effect model.

In the structural models constructed, we used the indices proposed by Wen et al. (2004) for evaluating model fit to assess the rationality of the models (Wen et al., 2004), as shown in Table 1.

**Table 1:** Organizational table of fitted indicators

Index	Judgment value
χ^2^/df	For reference only
RMSEA	< 0.05(Overfitting); < 0.08(Reasonable fitting)
SRMR	< 0.05(Overfitting); < 0.08(Reasonable fitting)
TLI	> 0.9
CFI	> 0.9

### Testing of common method bias

Due to the self-report nature of data collection in this study, there might be the presence of common method bias among different variables. To mitigate the issue of common method bias in survey research, we employed strategies such as anonymous surveys, reverse scoring items, and the inclusion of lie scale items for control. A factor analysis using Harman's single-factor test was conducted. In total, 10 factors with eigenvalues greater than 1 were extracted, accounting for 66.56% of the total variance. The first principal factor accounted for 39.26% of the variance, which is below the 40% critical threshold, indicating a reasonable control of common method bias (Zhou & Long, 2004).

### Descriptive statistics and correlation analysis

This study analyzed the differences in stress, rumination, social anxiety, and sleep quality based on household registration (urban vs. rural) and gender. There were significant differences in scores for stress, rumination, social anxiety, and sleep quality between male and female students (*p* < 0.01). No significant differences were found in stress, rumination, and sleep quality scores between urban and rural college students (*p* > 0.05), but there was a significant difference in social anxiety scores (*p* < 0.01), as shown in Table 2. Significant differences were observed in social anxiety and rumination among students from different academic years (*p* < 0.01), but no significant differences were found in stress and sleep quality scores (*p* > 0.05), as indicated in Table 3. The mean values, standard deviations, and correlation matrices for each variable are presented in Table 4.

**Table 2:** Differences in stress, sleep quality, social anxiety, and rumination thinking among college students of different genders and household registration

Dependent variable	Independent variable	F	Significance	t	Sig (two-tailed)
Stress	Genders	25.558	0.000	-2.776	0.006
Sleep Quality		0.054	0.815	-3.314	0.001
Social anxiety		3.706	0.054	-6.525	0.000
Rumination		15.653	0.000	-3.508	0.000
Stress	Household registration	0.281	0.596	1.577	0.115
Sleep Quality		7.990	0.005	-0.713	0.476
Social anxiety		2.432	0.119	3.016	0.003
Rumination		3.566	0.059	-0.629	0.529

*N* = 1653. Genders and Household registration are dummy variables, Male = 0, Female = 1, Rural = 0, Urban = 1

*p < 0.05, **p < 0.01, ***p < 0.001

**Table 3:** Differences in stress, sleep quality, social anxiety, and rumination among college students in different grades

Dependent variable		Independent variable	SS	DF	MS	F	Significance
Stress	intergroup	Grade	2026.04	5	405.208	1.468	0.197
	within a group		454515.732	1647	275.966		
Sleep Quality	intergroup		5.765	5	1.153	1.155	0.329
	within a group		1643.683	1647			
Social anxiety	intergroup		321.386	5	64.277	3.644	0.003
	within a group		29050.496	1647			
Rumination	intergroup		2637.482	5	527.496	3.074	0.009
	within a group		282613.518	1647			

*N* = 1653. Genders and Household registration are dummy variables, Male = 0, Female = 1, Rural = 0, Urban = 1

*p < 0.05, **p < 0.01, ***p < 0.001

**Table 4:** Mean, standard deviation and correlation coefficients of the variables

	1	2	3	4	5	6	7
1 Genders	1						
2 Age	-.119**	1					
3 Household registration	.004	-.065**	1				
4 Stress	.068**	-.021	-.039	1			
5 Sleep Quality	.081**	-.045	.018	.453**	1		
6 Social anxiety	.159**	-.094**	-.0740**	.530**	.428**	1	
7 Rumination	.086**	-.078**	.015	.659**	.531**	.564**	1
*M*				23.824	1.345	8.026	39.667
*SD*				16.624	0.999	4.217	13.140

*N* = 1653. Genders and Household registration are dummy variables, Male = 0, Female = 1, Rural = 0, Urban = 1

*p < 0.05, **p < 0.01, ***p < 0.001

### Construction and validation of structural equation models

In this study, due to the large number of items in the stress and rumination scales, direct modeling could affect the quality of indicator data and the true structure of the model. Therefore, we applied the item parceling method for modeling (Wen et al., 2018). Additionally, significant correlations were found between gender, age, household registration, and other variables in the study. Hence, gender, age, and household registration were included as control variables in the model.

**Fig. 1:**
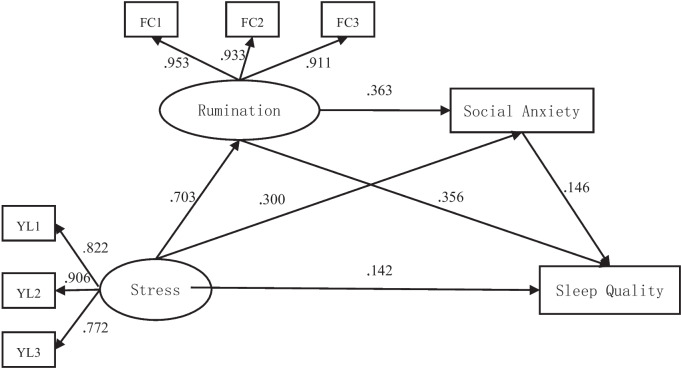
Multiple mediation model of rumination and social anxiety in the effect of stress on sleep quality

Firstly, we tested whether stress could directly predict sleep quality. The results showed that the model had a good fit index (as shown in Table 5), indicating that stress could significantly and positively predict sleep quality (β = 0.464, *p* < 0.001). Secondly, we constructed a structural equation model with stress as the independent variable, rumination and social anxiety as mediating variables, and sleep quality as the dependent variable. The results demonstrated that the model had a good fit index (as shown in Table 5), indicating that stress could positively predict rumination (β = 0.703, *p* < 0.001), rumination could positively predict social anxiety (β = 0.363, *p* < 0.001), and social anxiety could positively predict sleep quality (β = 0.146, *p* < 0.001), as detailed in Fig. 1.

**Table 5:** Model fit indicators organizer

Model	Index	Judgment value	Result
Stress → Sleep Quality	χ^2^/df	8.574	For reference only
	RMSEA	0.068	Reasonable fitting
	SRMR	0.040	Overfitting
	TLI	0.955	Reasonable fitting
	CFI	0.972	Reasonable fitting
Stress → Rumination → Social anxiety → Sleep Quality	χ^2^/df	9.244	For reference only
	RMSEA	0.071	Reasonable fitting
	SRMR	0.046	Overfitting
	TLI	0.958	Reasonable fitting
	CFI	0.970	Reasonable fitting

The chained mediating effects were tested using the Bootstrap resampling method with 1000 samples to calculate the 95% confidence interval. The results showed that the direct effect of stress on sleep quality was significant (β = 0.142, *p* < 0.001). Rumination and social anxiety mediated between stress and sleep quality, with a total mediating effect of 0.047. Specifically, the effect of Stress → Rumination → Sleep Quality was 0.036, the effect of Stress → Social anxiety → Sleep Quality was 0.006, and the effect of Stress → Rumination → Social anxiety → Sleep Quality was 0.005. The Bootstrap 95% confidence intervals for these indirect effects did not include zero, indicating that all three indirect effects were significant (as detailed in Table 6). Therefore, Hypotheses 1, 2, 3, and 4 in this study are supported.

**Table 6:** Bootstrap analysis of significance tests for mediated effects

Intermediary Path	Efficiency Value	95% Confidence Interval	
		Lower limit	Limit
Stress → Rumination → Sleep Quality	0.036	0.028	0.044
Stress → Social anxiety → Sleep Quality	0.006	0.004	0.010
Stress → Rumination → Social anxiety → Sleep Quality	0.005	0.003	0.008

## Discussion

The study found that stress, rumination, and social anxiety can significantly predict sleep quality, confirming Hypothesis 1. In contemporary society, people generally face pressures from various aspects such as academics, career development, and emotions, which negatively impact mental health and sleep (Ye et al., 2018; Lima et al., 2020; Zhao et al., 2021; Akerstedt et al., 2012). Especially at night, individuals may repetitively think about work-related issues or personal relationship challenges while trying to fall asleep. This rumination can lead to difficulty falling asleep, sleep interruptions, or early awakenings, and a prolonged poor mental state can cause increased social anxiety (Pillai et al., 2014). Poor sleep habits and low sleep quality can further lead to a range of physical health problems, such as cardiovascular diseases, impaired immune function, depression, and anxiety (Irwin, 2015). Therefore, reducing rumination in high-pressure situations can alleviate social anxiety, thereby improving sleep quality.

This study confirmed that rumination mediates between stress and sleep quality, consistent with existing research findings (Zhang et al., 2021), supporting Hypothesis 3. This can be explained from two perspectives: First, the theoretical model of stress cognition suggests that individuals go through stages of alarm, resistance, and exhaustion in response to stress (Friedman et al., 2005; Huang et al., 2016), and these stress responses, particularly in the exhaustion stage, cause significant changes in neurohormone levels, reducing sleep quality (Sun et al., 2014). Secondly, rumination, triggered by inducing factors and acting as a pre-sleep intrusive thought, continuously causes physiological and psychological arousal, preventing the onset of sleep (Brosschot et al., 2006; Pillai et al., 2014; Thomsen et al., 2003).

The study also found that social anxiety mediates between stress and sleep quality, confirming Hypothesis 2. Although there is limited research in this area, the Sensitivity Shift Theory (SST) suggests that inability to effectively cope with stress can lead to problems associated with social anxiety (Richey et al., 2019), which further impacts individuals, causing more stress and eventually leading to social anxiety disorders. The success or failure of individuals with social anxiety in responding to stress determines their willingness to continue facing stressors actively (Wright et al., 2010). The distressing emotions often cause them to rumination over negative social events, leading to physiological and emotional arousal and reduced sleep quality (Blote et al., 2021).

Furthermore, this study confirms that stress can predict sleep quality through the chained mediation of rumination and social anxiety, supporting Hypothesis 4. Response Style Theory posits that rumination formed in the process of coping with stress events, in turn, amplifies the impact of these events, increasing social anxiety and ultimately affecting sleep quality (Moulds et al., 2020; Robinson & Alloy, 2003). This chained mediation model also suggests to mental health educators that since college students are still developing their worldviews, values, and perspectives, and have limited cognitive levels and coping abilities, improving their mental health and sleep quality might be achieved by reducing their level of social anxiety, thereby interrupting the indirect impact of stress and rumination on sleep quality.

### Limitations and implications

This study has some limitations. Firstly, the issue of stress affecting sleep quality is common across different social groups, but our research subjects were university students. Extending the study to different demographic groups would better reflect real-world situations. Secondly, the measurements in this study were based on participants' self-reports, which are subjective experiences. While self-reports can provide valuable insights, they may not fully capture the reality. Future research could employ experimental methods or interviews to enhance the reliability of the findings.

Despite these limitations, this study contributes to the understanding of the internal mechanisms through which stress affects sleep quality, addressing some gaps in previous research. The study enriches the field's understanding of stress cognition theoretical models (Friedman et al., 2005), Response Style Theory (Moulds et al., 2020; Robinson & Alloy, 2003), and Sensitivity Shift Theory (Richey et al., 2019). In the context of psychological interventions in schools, in addition to offering stress reduction training such as meditation, mindfulness, and diaphragmatic breathing, schools can also teach students positive thinking, proactive actions, and help them develop a healthy and positive mindset to alleviate their social anxiety.

## Conclusion

The research discovered that stress can not only directly and positively predict sleep quality but also independently mediate and positively predict sleep quality through rumination and social anxiety. In addition, rumination and social anxiety play a chained mediating role between stress and sleep quality. This indicates that the mechanisms by which stress affects sleep quality are complex. We cannot simply assume that stress directly impacts sleep quality; in reality, stress more significantly affects sleep quality through rumination and social anxiety.Therefore, in addition to helping individuals reduce stress through some commonly used psychological intervention pathways, we should pay more attention to how to help help helpers reduce rumination thinking and social anxiety, so as to reduce the impact of stress on sleep quality, which is of great significance to improve the sleep quality of individuals.

## Data Availability

The datasets used and/or analysed during the current study are available from the corresponding author on reasonable request.
